# How do general practitioners handle couple relationship problems in consultations? A focus group study

**DOI:** 10.1093/fampra/cmac010

**Published:** 2022-02-18

**Authors:** Siri D Berge, Mette Brekke, Eivind Meland, Thomas Mildestvedt

**Affiliations:** Department of Global Public Health and Primary Care, University of Bergen, Bergen, Norway; General Practice Research Unit, Department of General Practice, Institute of Health and Society, University of Oslo, Oslo, Norway; Department of Global Public Health and Primary Care, University of Bergen, Bergen, Norway; Department of Global Public Health and Primary Care, University of Bergen, Bergen, Norway

**Keywords:** couples therapy, family relations, general practice, general practitioners, marriage, primary health care

## Abstract

**Background:**

Couple relationship problems are common and associated with health problems. The aim of this study was to explore general practitioners’ (GPs’) experiences, expectations, and educational needs when dealing with couple relationship problems in consultations.

**Methods:**

We conducted an exploratory qualitative study by carrying out 3 semistructured focus group interviews with 18 GPs. We used systematic text condensation for the analyses.

**Results:**

Participants shared their experiences of handling couple relationship problems in consultations. Three main themes emerged: (i) pragmatic case-finding: golden opportunities to reveal patients’ couple relationship problems; (ii) conceptual and role confusion; (iii) professional competence and personal experience. While issues in relationships could serve as an explanation for relevant clinical problems, some GPs questioned whether relationship issues are strictly medical. All participants had engaged in individual supportive therapy, but none saw themselves as therapists. The interviews revealed that an individual supportive focus might lead to a consolidation of 1 partner’s view, rather than challenging their position. Long-term doctor–patient relationships made it easier to talk about these issues.

**Conclusions:**

This study revealed several paradoxes. GPs are confident in offering individual supportive therapy for couple relationship issues but should be aware of substantial pitfalls such as side-taking and constraining change. Despite dealing with relationship problems, GPs do not see themselves as therapists. They use professional and personal experience but would benefit from increasing their skills in cognitive restructuring promoting behavioural flexibility facing relationship problems.

**Key messages**

- Couple relationship problems are often presented as health problems.
- GPs take a holistic approach in dealing with couple relationship problems.
- GPs face a dilemma between offering empathic support and challenging their patients.
- Doctors lack tools to assess couple relationships and offer counselling.
- Doctor–patient continuity gives GPs confidence in handling relationship problems.

## Background

Couple relationship satisfaction is related to physical and mental health and longevity,^1^ and is more important for life quality than job satisfaction and friendships.^2^ Children’s physical and mental health are also affected by their parents’ relationship quality.^3^

Less than half of individuals experiencing divorce seek marital therapy before separation.^4^ Couples therapy and couple relationship education (CRE) are the main sources of help-seeking for couples. Their efficacy is well known but both reach a limited and resourceful section of the population.^4^ Brief interventions (BIs) are instructions in daily interactions between the partners which are uncomplicated to implement. They are as effective as traditional CRE and easily accessible.^5^ Daily gratitude or appreciation, doing activities together, and turning towards bids for connection are examples of BIs.^5^ A bid for connection is anything you or your partner does to connect with the other. Examples of bids are sending a text, giving a kiss, stopping what you do to greet your partner at the door, or sharing their day.

A mental health problem is brought up in 1 of 4 general practitioner (GP) consultations. This includes relationship problems in general, and couple relationship problems in particular.^6^ Family and partner conflicts are a significant proportion of consultations with psychosocial themes.^7^

In a Norwegian survey, 1 in 4 patients had talked to their GP about couple relationship issues, one-third wanted to talk with their GP, and almost half of the patients wanted their GP to take an interest in their couple relationship.^8^ Another Norwegian study on expectations from patients and GPs regarding health care-seeking behaviour showed similar results. Almost one-third of patients thought that most patients would arrange to see their GP when having relationship problems.^9^

GPs do not feel competent to counsel relationship problems in general and need additional training regarding relationship management.^10^ On the other hand, with adequate training they could include couples counselling in their practice, particularly to identify couples in crisis, and to provide preventive strategies to improve marital satisfaction.^11^ A study on marital satisfaction from American primary health care proposed that a 1-item question with a visual analogue scale (VAS) could be as useful as a 32-item screening tool in primary care.^12^

GPs’ experiences of dealing with couple relationship problems among their patients are largely unknown. Do they expect to have a role in handling such problems, and if so, do they feel competent to do it?

The aim of this study was to explore GPs’ experiences, expectations, and educational needs when dealing with couple relationship problems in consultations.

## Methods

### Study design and recruitment

We carried out an exploratory qualitative study using a sample of GPs from Norway. We used social media and our professional network to recruit participants. We sought maximum variation in terms of sex, age, urban and rural location, and years in practice. Twenty-six GPs were approached, and 18 accepted the invitation to participate.

A focus group design was chosen to produce data based on the interactions between the participants that would not be possible to get from individual interviews.^13^ The group dynamic often contributes to a wider range of perspectives and ideas. Experiences from other participants often inspire new associations leading to richer information from focus groups compared to the sum of individual interviews. Some studies have found that individual interviews produce more sensitive themes than focus groups, while others have found opposite.^14^

### Data collection

We conducted 3 semistructured focus group interviews between June and October 2020. The interviews were conducted and video-recorded by 2 researchers (SDB and TM) using the digital platform Zoom because of the Covid-19 pandemic. Each interview lasted 90 min. SDB facilitated the groups, while TM observed. Both researchers took field notes during the interviews and discussed their immediate impressions after each interview ended. We developed and used a semistructured and flexible interview guide as shown in Box 1.

Box 1. **Interview guide used in the focus group interviews of Norwegian GPs about how they handle couple relationship problems in consultations (2020).**

Introduction: brief presentation of the research project

- What experience do you have handling couple relationship problems in your practice?
  - Any examples?
  - What do the patients who talk about their couple relationships in the consultation want?
  - What do they expect from the doctor?
- In what situations do you feel talking about couple relationship problems are relevant?
  - Do you have any examples of particular situations where you felt talking about couple relationship was relevant?
  - Do you have the opposite experience—where talking about couple relationship issues appeared to be inappropriate? What happened?
- Why do you think it is useful for patients to see their GP when experiencing couple relationship problems?
  - Do you have examples of situations where you think the patients felt it was useful?
- What experience do you have handling couple relationship problems involving children?
  - Do you have examples of situations whereas talking about children has been an issue while talking with patients about couple relationship problems?
  - Do you have examples of situations where you have talked about child custody or childcare?
  - What role should the GP have regarding children and couple relationship problems in your opinion?
- What role do you want couple relationship problems to have in your practice?
  - What do you expect from yourself?
- Do you need any increased skills about managing couple relationship problems?
  - What kind?
  - In which way?

Each group consisted of 6 GPs mainly with an established relation to each other. The first group consisted mostly of GPs specializing in general practice and was well established with regularly meetings. In the second group, all participants were approved specialists with a long experience from general practice. The last group consisted of 5 experienced GP specialists and 1 in GP training.

Sample size should be guided by information power as described by Malterud,^15^ and this is typically met by 2–5 focus groups.^16^

### Data analysis

The video files were transcribed verbatim by SDB and imported into the qualitative analysis software NVIVO12. We used systematic text condensation for the analysis.^17^ The analysis was explorative and not based on any existing theoretical framework, although the researchers are GPs and take a biopsychosocial approach in their respective practices. All 4 authors participated in the analysis. First, we established an overview of the data with an open mind to get a general impression. Then we agreed on preliminary themes for organizing the data and identified meaning units sorted into code groups. We developed codes for each theme in an iterative process until agreeing on the final themes for describing the data (Table 1). The third step of the analysis was to merge the meaning units and reduce the content of every subgroup into condensates. At last, we recontextualized the data and developed descriptions and concepts.

**Table 1.** Categories and subcategories identified as part of the systematic text condensation and most representative quotes from the focus group interviews with Norwegian GPs about how they handle couple relationship problems in consultations (2020).

Categories	Subcategories	Quotes	GP characteristics
Pragmatic case-finding: golden opportunities to reveal patients’ couple relationship problems	Patients’ presented problems	*It is presented as something different from what it is.*	GP9, male, aged >60 years, >20 years’ practice experience
	Children as a pathway to talk about relationship problems between the parents	*Sometimes you get information about difficult domestic situations through children who struggle.*	GP7, male, aged >60 years, >20 years’ practice experience
	Checkpoints	*It is an opportunity to address the topic at the routine control six weeks after birth.*	GP7, male, aged >60 years, >20 years’ practice experience
Conceptual and role confusion	Not strictly medical	*It is not directly medical, but it could have rather significant effect on the patient’s wellbeing.*	GP1, female, aged <40 years, 5–9 years’ practice experience
	Lack of education	*I had had some conversations regarding couple relationships, strained couple relationships over a period. And I think that I, as a GP and with my education, do not really have the background to offer couples therapy. And therefore, I find it quite challenging.*	GP13, female, aged 40–49 years, 10–19 years’ practice experience
	Supportive therapy as default method not acknowledged as therapy	*I do not think they expect me to be their therapist. But some are coming just to think out loud together with someone impartial. And partly to find a way through chaos.*	GP17, male, aged 40–49 years, 10–19 years’ practice experience
Professional competence and personal experience	Competence in cognitive restructuring.	*I do lots of conversations. If it is cognitive therapy or about life and death.*	GP15, female, aged 50–59 years, >20 years’ practice experience
	Long-term doctor–patient relationships	*New issues emerge when you have been working in the same practice for a long time. You have followed them [the patients] through life.*	GP13, female, aged 40–49 years, 10–19 years’ practice experience

## Results

Eighteen GPs from 16 different practices participated in 3 focus groups with 6 participants in each group. Characteristics of the participants are presented in Table 2.

**Table 2.** Demographic variables of the 18 participants attending a focus group study about Norwegian GPs’ experience from talking with patients about couple relationship problems (2020).

Variables	*N*	Missing	%	Median	Min	Max
Age	18	0		43.5	32	71
Sex	18	0				
Women	10		55.6			
Men	8		44.4			
List size^a^	18	0		1,060	600	1,300
Number of GPs at the office^b^	18	0		5	2	10
Years in a GP practice	18	0		10.5	4	40
Approved general practice specialist	18	0				
Yes	12		66.7			
No	6		33.3			
Urban/rural	18	0				
Urban	12		66.7			
Rural	6		33.3			

The number of patients each GP is responsible for.

GP, general practitioner.

All participants reported numerous experiences of handling couple relationship problems in their practice. None of the physicians had a special interest in couple relationship problems, nor were they trained in couples counselling techniques. Three main themes emerged: (i) pragmatic case-finding: golden opportunities to reveal patients’ couple relationship problems; (ii) conceptual and role confusion; (iii) professional competence and personal experience.

### Pragmatic case-finding: golden opportunities to reveal patients’ couple relationship problems

The participants rarely identified couple relationship problems as the reason for a patient encounter, but these problems emerged during a holistic approach to the patient. They highlighted that GPs should ask about their patients’ couple relationships more often, invite patients to talk about their relationship issues to reveal problems earlier, and be able to do preventive work. Golden opportunities to ask about patients’ relationships as a way of revealing couple relationship problems were mentioned.

The patients’ presenting problems were, according to the GPs, often pain conditions, tiredness, or troubled sleep. There are different approaches to such conditions, and the GPs made a point of looking for underlying causes, and being open to biomedical, psychological, social, and existential explanations, including couple relationship problems. At the same time, they often failed to address this issue and often focussed only on the presenting problem itself. The GPs had the impression that patients were often in need of a period of sick leave when going through a divorce.

Asking about social networks and couple relationships in patients with a chronic or severe illness was emphasized. Such illnesses included psychiatric problems, unexplained symptoms, or sexually transmitted infections. Elderly patients were especially identified as vulnerable to couple relationship problems, because of well-known risk factors including chronic disease and cognitive impairment which are more common in this group. The doctors reported elderly patients who were unsure whether it was appropriate for them to start a new relationship when their spouse did not recognize them anymore. They also reported older people bringing up couple relationship issues such as domestic violence, sexual problems, and the experience of divorced patients being old and alone. Hence, routine check-ups for patients with chronic disease and/or elderly patients were seen as golden opportunities to talk about the patients’ couple relationships.

Addressing sexual health issues with both genders and at any age was a part of couple relationship problems the participants felt competent about. There was a tendency for female doctors to more frequently experience this issue being brought up by their female patients, while male participants more often heard their male patients’ queries about sexual problems. Everyone considered this an obvious task for a GP and a golden opportunity to ask more questions about the couple relationship.

For younger patients, the importance of being an advocate for the children in a family and promoting awareness of how the children might be feeling was highlighted. It was emphasized how parental conflicts affect children. Some had invited parents to reflect on how their children would be affected by their parents’ relationship problems and used the children as a pathway to talk about the patients’ relationships.

Pregnancy check-ups is a part of a GPs regular work in Norway. Most pregnant women are seeing their GP during pregnancy, and 6 weeks after giving birth. Hence, pregnancy check-ups were seen as a golden opportunity to ask about the patient’s couple relationship.

The first encounter with a new patient is often utilized to get to know him or her by asking about medical history and risk factors such as smoking and other lifestyle issues. This was highlighted as a golden opportunity to ask about marital status and 1 or more questions about the patient’s couple relationship.

### Conceptual and role confusion

The GPs discussed whether couple relationship problems should be an issue of concern in consultations. We revealed an uncertainty, in that some GPs wondered if relationship problems were “medicine” or “just life,” as a way of asking if this kind of issues are something a GP should devote valuable consultation time to. Others stated that all relationships, especially couple relationships, were important to health. These informants also emphasized the importance of a holistic approach to their patients’ symptoms and life challenges.

Some of the less experienced doctors said that they would embrace this issue, in addition to all the other tasks in general practice, if research could convince them that relational problems are indeed important for health. During the interview, the less experienced GPs in general seemed to have a stronger biomedical focus and less holistic perspectives on patients’ medical problems and health.

When GPs were behind schedule, it was common for them not to ask about couple relationship problems, despite their awareness of taking the recommended holistic approach. One way to deal with time limitations was to acknowledge the problem and schedule a lengthier consultation towards the end of a workday. The option of referring couples with problems to the Family Welfare Service were appreciated. Several participants called for tools to assess relationships and to help them offer counselling.

Everyone had experience of talking with patients individually about their couple relationship problems, but only a few had talked with patients as a couple. There was a common opinion that GPs should not act as psychotherapists practicing couples therapy unless they were especially interested and qualified. However, all the GPs offered support to their patients, and most of them pointed out that this is an important part of the GP’s holistic approach. Paradoxically, the GPs did not see this support as therapy.

The GPs had the impression that patients expected them to be supportive—and they were aware that this could be a problem when both partners in a couple attended the same physician. Some reported difficulties in being impartial with both partners, to avoid the appearance of taking sides. An individual supportive focus could lead to the consolidation of 1 partner’s point of view, rather than challenging their position and helping them to see their problems from a more neutral perspective.

Others saw it as an advantage to be both partners’ GP because it made it easier to see the whole picture and challenge their views on the situation. Some emphasized that in medical education doctors learn communication skills useful for individual consultations, but not systemic counselling skills.

### Professional competence and personal experience

The GPs in the focus groups had different amounts of training in psychotherapy. Several of the participants had competence in cognitive therapy techniques and restructuring. They used this competence mostly in individual consultations, but a few of the participants had tried dyadic consultations with couples. This cognitive approach could both challenge and equip patients. It was described as a useful tool that could contribute in a more constructive way dealing with relationship problems.

A continuous doctor–patient relationship was described as important in enabling patients to talk with their GP about couple relationship issues. The participants had the impression that frequent changes of GPs made patients feel less safe in opening up about vulnerable issues and feelings of shame could become a hampering factor. Shame was brought up by the participants as both a hindrance and a facilitator, and they experienced that it affected whether their patients chose to talk with them about their couple relationships. Sometimes, according to the participating GPs, shame prevented patients from confiding in friends and encouraged them to open up to their GP instead.

The experienced doctors felt more comfortable talking about couple relationships compared to younger doctors. The most experienced emphasized that it was mainly their professional experience, but also, to a certain extent, their personal experience that qualified them to support patients with couple relationship problems. Patients brought the subject up more often now than they could recall from earlier in their GP career.

One participant mentioned that negative personal experiences could activate vulnerable emotions when talking with patients about couple relationship problems.

The GPs saw themselves as a medical facility easy to contact and attend, and they wanted to be supportive and understanding. They often took on the role of being a moderator, a sparring partner, or, as one of the participants described it, a dumping ground for negative emotions. Others saw themselves as an impartial and independent third party who created a safe zone for patients to talk.

The informants felt that patients expected them to take a holistic approach, and to be competent in talking about most topics. One of the GPs stated that patients do not expect a solution, just someone impartial to talk to.

## Discussion

### Summary of key findings

The participating GPs reported numerous experiences of handling couple relationship problems in their practice. We identified 3 main themes: (i) pragmatic case-finding: golden opportunities to reveal patients’ couple relationship problems; (ii) conceptual and role confusion; (iii) professional competence and personal experience.

### Comparison with existing literature

Pragmatic case-finding is a well-known strategy in general practice to reveal health problems among patients.^18^ A study on how GPs talk about alcohol found that the duality between shame and normality, time constraints and a need for structure were the most important barriers.^19^ This correlates with our findings regarding couple relationship issues: shame and time constraints were barriers, while routine check-ups and pregnancy check-ups were seen as opportunities to bring up the topic.

The biopsychosocial model^20^ is understood as the leading framework for general practice in recent decades,^21^ but it has been claimed that this is more of an academic model than actually fully implemented in clinical general practice.^22^ Our study revealed that a dilemma still exists between being a doctor who is responsible for finding biomedical explanations for symptoms and being a doctor who experiences that symptoms frequently need to be explained by the integration of relational and psychological factors. This is in line with knowledge that chronic shame, relational problems, and negative emotions cause prolonged stress in the body, which influences physiological systems, such as the immune and cardiovascular systems.^1,23^ There was a tendency that less experienced GPs had a stronger biomedical perspective than their more experienced colleagues. This might reflect that medical education still has a strong biomedical focus.

The GPs in our study were not comfortable with the label of therapist, as in psychotherapist or couples therapist, even though acting as a doctor in relationship with a patient is considered a therapeutic act.^24^ Is there a conflict between being a GP and a therapist? A therapeutic relationship between a physician and a patient is important.^25^ Many GPs, including some of our participants, are educated in cognitive therapy,^26^ but they seldom talked with patients as couples, rather they had consultations with 1 patient at a time. Supportive therapy is widely used by GPs,^27^ and the most important element is the therapeutic alliance.^28^ It is possible that supportive therapy is so deeply integrated into the GPs’ professional skills that they are not aware that they offer therapy. By this conceptual confusion, a “blind spot” might be introduced in their clinical practice. Supportive consultations are by many considered as therapy.^28^

Individual counselling for couple relationship problems is known to have several pitfalls, such as constraining change, therapist side-taking, and inaccurate assessments based on individual client reports.^29^ The GPs we interviewed were aware of the risk of taking sides. Applying individual supportive therapy on couple relationship problems generates a risk of reinforcing 1 patient’s negative emotions about their partner and could indirectly increase the risk of divorce.^29^ When engaged in supportive therapy, it is important to challenge, rather than just please, the patient. Increasing skills in systemic approaches could be beneficial in this context.^30^ Nevertheless, the effect of such interventions depends on the GP’s ability to enhance function while empowering the patient, not consolidating the patient’s problems, and making them pitiable.

With the international campaign of overdiagnosis named “Too much medicine”^31^ in mind, GPs should be aware of the risk of medicalization of normal life events such as couple relationship problems. On the other hand, overdiagnosis is defined as the diagnosis of a condition that, if unrecognized, would not cause symptoms or harm a patient during their lifetime.^31^ It is well known that couple relationship problems can cause various health problems and are an independent risk factor for disease.

Our participants wanted tools to assess and engage in BIs. Assessing tools are available but require further validation in general practice, such as 1 or a few questions to identify patients with couple relationship problems.^12,32^ GPs could easily teach BIs.^5^ This could even save consultation time in a long term because patients get a manageable tool. Giving psychoeducation about typical relational patterns could increase the patient’s understanding of the problem. Challenging the patient to take his or her partner’s perspective could increase the understanding of the situation even more.^5,33^

Continuity in clinical relationships is an important tool for the GP, is vital in person-centred care, and is recently shown to be significantly associated with reduced need for out-of-hours services, acute hospital admissions, and mortality in a dose-dependent way.^34^ Since 2001, every inhabitant in Norway can actively choose their own regular GP, and more than half state that it is important to keep a GP they already know.^34^ A continuous relationship between a regular GP and a patient will most often be of good quality because the patients actively choose their own regular GP. The experienced GPs with longer doctor–patient relationships more often talked about relational problems with their patients. The continuity of the GP–patient relationship is nowadays threatened because of the current crisis in general practice in several countries.^35–37^

To be a good doctor, academic and professional skills are important, but equally important are the doctor’s personal qualities.^38^ Our informants lacked professional training in couples counselling but underlined the professional and personal experience they developed over time and saw this as a useful tool in their toolbox when helping patients with couple relationship problems. However, our data revealed large variations in doctors’ own understanding of their role: from being a dumping ground to engaging in cognitive restructuring.

### Strengths and limitations

The internal validity was strengthened by the participants’ and the researchers’ common background as GPs. This enhanced the mutual understanding of which questions the discussion was going to answer and facilitated a safe place for the conversation because the participants shared common experiences from being a GP.

The GPs felt secure enough to discuss topics that exposed their own vulnerabilities. They commented on each other’s experiences and expressed disagreements during the interviews. The conversations flowed easily even though the interviews were conducted digitally. The interviewer did not have to ask several questions, because the participants had productive conversations where 1 statement built upon another. The participants mostly knew each other. Only 1 GP did not know the other group members but did still engage in the conversation as much as the rest of the group members. The talking time was fairly equally split between the members in all 3 focus groups, and the interviewers asked questions to clarify the participants’ statements to reveal different voices and different opinions in the focus groups. The interviewers were aware of the risk of peer pressure in a focus group, and intentionally ensured that all members of the group were allowed to be honest and open about their own perspectives.

It is important to aim for reflexivity and to be aware of the researchers’ positions and experiences, and how these could affect the study.^39^ All 4 authors are experienced GPs with a special interest in couple relationship issues. SDB and TM who accomplished the focus group interviews perform CRE outside their GP practice. MB and EM are former experienced GPs and educated in systemic therapy. The authors’ background as GPs is a strength when interviewing other GPs, since it is easier to understand their working context and which limitations they meet in everyday clinical practice. On the other hand, there is always a risk of missing out an outside perspective.

A focus group design is well suited for studying experiences and attitudes.^13^ The external validity was strengthened by a varied and adequate study sample with a heterogeneous group of participants in terms of gender, age, years of experience, and location of practices (urban/rural). A relevant and strategic selection of participants are important for external validity and strengthen the transferability of the findings.^15^ We used information power to decide the sample size. Information power can be divided into 5 items: narrow or broad study aim, dense or sparse sample specificity, applied established theory or not, strong or weak quality of dialogue, and case or cross-case analysis strategy.^15^ Our study aim was to explore how GPs handle couple relationship problems, which is narrower. The setting is familiar to most GPs, since 25% of the patients have talked about their couple relationship problems with their GP.^8^ The sample specificity was dense because the participants held characteristics highly specific for the study aim. The quality of the dialogues was strong with clear communication among the focus group members and between the participants and the researchers. At last we did an exploratory cross-case analysis which requires more participants than a case analysis focussing on in-depth analysis of narratives or details from a few selected participants.^15^

### Implications of findings for research

GPs want to support their patients even though they are constrained by time. They would benefit from tools for pragmatic case-finding, to do assessments, and increasing their counselling competence. Further research should focus on testing and validating simple tools that could be useful in a busy practice. Such tools could be 1 or a few questions to identify patients with couple relationship problems, the implementation of BIs, providing psychoeducation, or challenge a patient to take his or her partner’s perspective on the problem.

It would also be useful to explore patients’ experiences from talking to their doctors about their couple relationship before developing and implementing tools.

## Conclusion

This study revealed several paradoxes. GPs are confident in offering individual supportive therapy for couple relationship issues but should be aware of substantial pitfalls such as side-taking and constraining change. Despite dealing with relationship problems, GPs do not see themselves as therapists. They use professional and personal experience but would benefit from increasing their skills in cognitive restructuring promoting behavioural flexibility facing relationship problems.

## Data Availability

The data underlying this article cannot be shared publicly for ethical reasons and the privacy of the individuals who participated in the study. The data will be shared on reasonable request to the corresponding author.
